# Incidence of pathologically positive lymph node in clinico-radiologic T4b oral squamous cell carcinoma: a meta-analysis

**DOI:** 10.3332/ecancer.2026.2138

**Published:** 2026-06-02

**Authors:** Sameer Pandey, Sooraj Pillai, Megha Punetha, Santhosh Rao

**Affiliations:** 1 Government Medical College Haldwani, Nainital, Haldwani, Uttarakhand 263139, India; 2 Oral and Maxillofacial Surgery, Swami Vivekanand Subharti University, Meerut, Uttar Pradesh 250005, India; 3 Soban Singh Jeena Government Institute of Medical Sciences and Research, Almora, Uttarakhand 263601, India; 4 All India Institute of Medical Sciences Raipur, Raipur, Chhattisgarh 492099, India; a https://orcid.org/0000-0002-7070-8237; b https://orcid.org/0009-0009-2310-2329; c https://orcid.org/0000-0002-2448-9854; d https://orcid.org/0000-0003-0274-7442

**Keywords:** oral cancer, metastasis, lymph node, carcinoma, head and neck

## Abstract

**Background:**

Clinico-radiologic T4b oral squamous cell carcinoma (OSCC) lesions form the most advanced cohort of oral malignancy. Survival outcomes depend on major prognostic determinants, such as nodal status. The anatomic extent of disease determines triage for curative-intent surgery.

**Purpose:**

The purpose of the meta-analysis was to find the incidence of lymph-node metastasis and estimate the survival outcomes of patients with cT4b OSCC treated with curative intent surgery.

**Data sources:**

PubMed, Scopus, Ovid, Cochrane Library, Embase and ClinicalKey databases were searched from July 2005 to July 2024. A manual search of speciality journals was conducted from July 2005 to July 2024, without any language restriction and reporting only human studies.

**Study selection:**

All randomised controlled trials (RCTs), non-RCTs, prospective, retrospective studies based on cT4b OSCC of all subsites except tongue were included in the review.

**Methods for selecting the study:**

Studies were reviewed by two reviewers, and a third reviewer resolved any disagreements.

**Data extraction and synthesis:**

The extracted data were recorded in a pre-piloted Excel sheet. The synthesis of results was performed using MedCalc version 23.0.6 software.

**Main outcomes and measures:**

A PRISMA-guided systematic review was conducted. Data on the incidence of N0 disease, the number of cases with clear surgical margins, overall survival and disease-free survival were recorded. Based on data homogeneity, a proportional meta-analysis was performed to evaluate the weighted incidence of N0 disease.

**Results:**

Eleven studies were shortlisted for data extraction. Due to heterogeneous surgical techniques and criteria for surgical margins and survival duration, outcomes were analysed only for the incidence of N0 disease. Results suggested that 40.58% of patients had pathologically N0 disease. There was statistically significant heterogeneity (I2 = 93%).

**Conclusion and relevance:**

Among patients with cT4b OSCC, 40.58% have negative neck disease, with carefully evaluated information on anatomic spread, a curative intent surgical treatment plan can be designed.

## Introduction

Oral squamous cell carcinoma (OSCC) continues to be a significant oncologic burden in India, with up to 64% [1] of patients presenting in stage IV disease. A US-based National Cancer Database study reported a 55% incidence of locally advanced head and neck malignancy [2]. The anatomical spread and the surgical resectability of the tumour have guided the treatment algorithm for locally advanced OSCC. Lymphatic spread of disease is a significant predictor of survival in OSCC [3].

Locally advanced OSCC is usually a term denoted to T3-T4 lesions. In contrast, there has been a broad consensus regarding the surgical management of T3 and T4a lesions. However, T4b lesions have been grouped with unresectable tumours in the NCCN 2025 version guidelines [4]. These tumours are defined as involving the masticator space or skull base/pterygoid plates or internal carotid artery encasement. However, the tumours of the maxillary sinus with skull base and infratemporal fossa (ITF) involvement have been grouped as cT4a in TNM for maxillary sinus malignancies [5]. The recently updated TNM 9th edition [5] Classification has removed the terminology of ‘locally advanced’ or ‘very advanced’ malignancy. However, it still keeps all the oral cavity squamous cell carcinoma (SCC) involving the masticator space in T4b [5] same as in previous TNM 6th, 7th and 8th edition.

Triage for surgical treatment of very advanced disease (cT4b) depends on surgical resectability, which refers to the technical ability to obtain clear surgical margins [6]. Compartment resection has become a standard surgical approach to address T4b OSCC, with a well-established oncologic safety [7].

The prognosis of patients with OSCC and their survival outcomes can be predicted by lymph node disease status.

Reclassification of cT4b OSCC has been consistently discussed since the introduction of compartment resection in surgical management. While a series of prospective and retrospective studies have been conducted, only a few systematic reviews of the literature and meta-analyses have been published on the subject. The systematic review aims to identify the available literature and meta-analyse published data to estimate the critical prognostic determinants, i.e., Lymph node metastasis in patients with T4b OSCC and to generate better guidelines.

## Materials and methods

A PRISMA [8] a guided systematic review protocol was registered in PROSPERO (Reg. No. CRD42021230116). An electronic database search was performed in PubMed, Cochrane Library, Embase, ClinicalKey, Ovid Discovery and Scopus from 2005 to July 2024.

Manual searches of journals such as Laryngoscope, Oral Oncology, the International Journal of Oral and Maxillofacial Surgery and Journal of Oral and Maxillofacial Surgery were conducted for published issues from 2005 until July 2024. The references of shortlisted articles were also examined to identify additional studies. MeSH terms and an advanced search strategy (Table 1) were utilised to identify studies based on the following Population, Intervention, Comparator, Outcomes (PICOs) criteria.

Research questions:

a. What is the proportion of cT4b OSCC patients without lymphatic metastasis?
b. What is the overall disease-free survival (DFS) and overall survival (OS) rate in supra-notch T4b SCC patients treated surgically?

### Outcomes

Primary: Incidence of lymph node metastasis, DFS and OS.

Secondary: Disease spread anatomical indication for upfront curative intent surgery.

### Study designs

This review included randomised controlled trials (RCTs), non-randomised controlled trials (non-RCTs), prospective studies, retrospective studies and case series of more than ten cases. No language restrictions were applied in the review. The studies, which were based on specific disease sites (OSCC of subsites, including buccal mucosa, Gingivobuccal sulcus and maxillary alveolus) with clinical staging of T4b, were included in the review. Only studies reporting curative intent surgery with compartment resection or specimen-driven resection were included in the review.

### Data extraction measures

A pre-piloted Excel sheet was used to record data under the following sections: author, total number of patients treated, total DFS, OS, percentage of clear margins, pathologically N0 nodes and clear margins.

### Risk of bias and critical appraisal of included studies

Robins-I and ROB-2 tools from Cochrane were utilised to assess the risk of bias. A risk of bias traffic plot and a risk of bias summary were produced using the Robvis tool. Studies were appraised for bias related to the outcome examined in the meta-analysis. SP and SoP participated in the risk of bias assessment and any disagreements were resolved by MP.

### Strategy of statistical analysis and synthesis of results

The pooled data on the proportion of patients treated surgically with pathologically free lymph nodes were used to perform a proportional meta-analysis using MedCalc version 23.0.6 [9].

A similar meta-analysis was conducted for the proportion of patients with 5-year DFS and OS rates. Given the expected clinical and methodological heterogeneity among the included studies, a random-effects model was used to generate pooled estimates, as it offers a more conservative summary by accounting for between-study variability.

Publication bias was evaluated by using Egger’s test and Kendall’s test and represented through a funnel plot.

Heterogeneity was quantified using *I*^2^ test, where *I*^2^ values between 0%–40% and 30%–50% were considered moderate. 50%–90% substantial heterogeneity, 75%–100% considerable heterogeneity.

## Results

**Figure 1.**
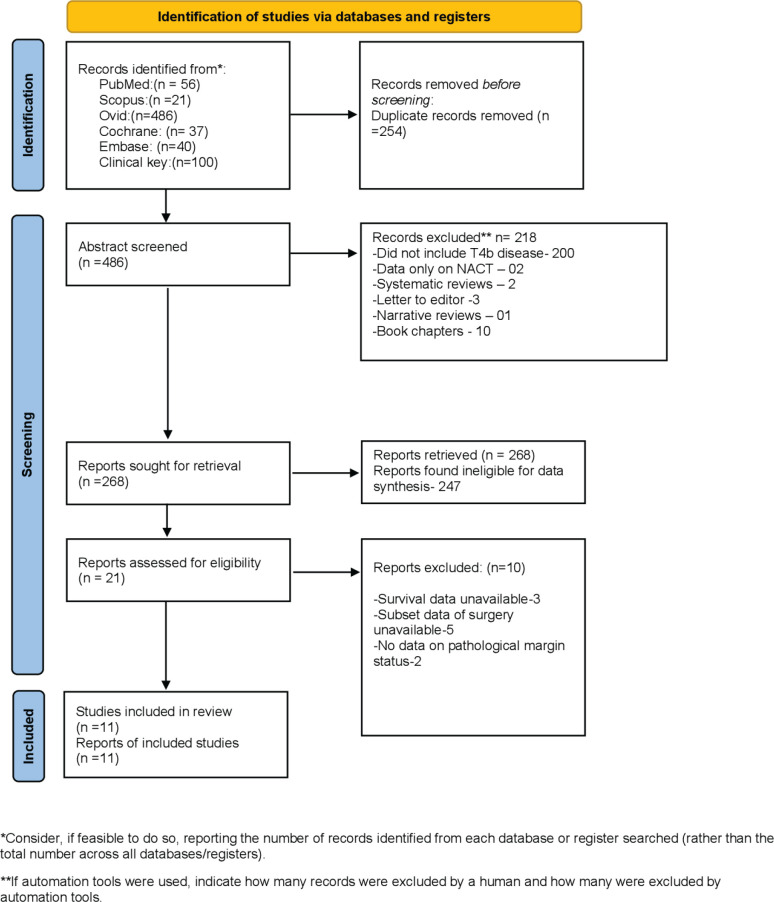
PRISMA flow chart.

Results of search strategy: Database searches identified (Table 1) a total of 740 articles for de-duplication and abstract screening. After de-duplication and abstract screening, 220 articles were sought for full-text screening. Eleven articles [10–20] were shortlisted for final data analysis and inclusion in meta-analysis (Table 2). There were 1,229 patients of cT4b OSCC treated with curative intent surgery (Figure 1).

### Data analysis

**Figure 2.**
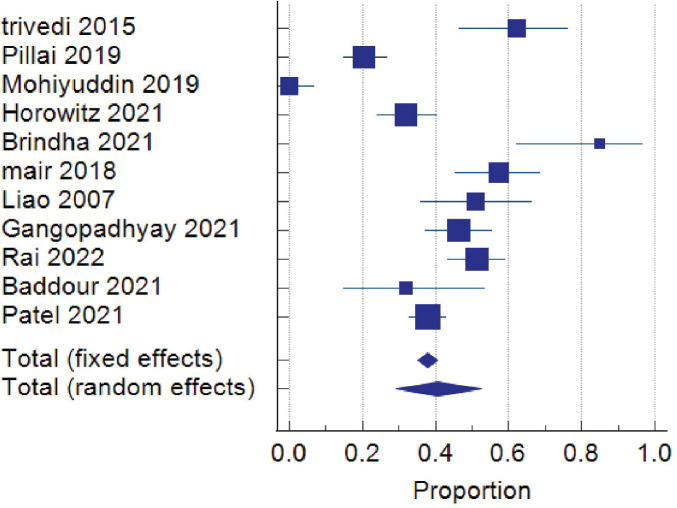
Forest plot of proportional meta-analysis (incidence of N0 in cT4b disease).

**Figure 3.**
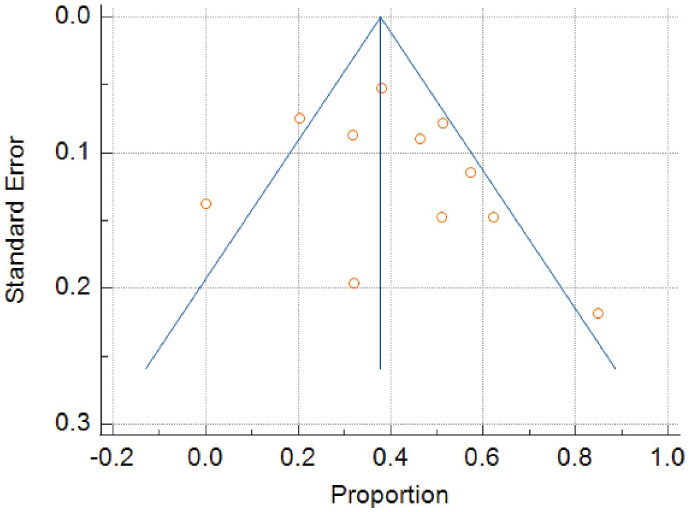
Funnel plot -representing risk of publication bias.

A proportional meta-analysis of the number of patients treated surgically with pathologically negative nodes revealed that 40.58% patients had no lymphatic disease spread (Figure 2) (*I*^2^ = 93%, random effect model). The funnel plot (Figure 3) showed a relatively wide distribution of studies, indicating heterogeneity.

An attempt was also made to extract hazard ratios (HRs) and 95% confidence intervals from Kaplan-Meier curves and reported survival data to enable a time-to-event meta-analysis. However, due to inconsistent reporting formats, lack of HRs in several studies and significant heterogeneity in survival endpoints (DFS, OS at varying time points), pooling HR-based outcomes was not feasible. Instead, survival outcomes were summarised narratively. Table 3 presents the reported survival rates in patients with supra-notch T4b disease. Three years of DFS have been reported from 39% to 51% [10, 13, 17–19]. OS at 5 years has been reported as 14% to 51% [10, 13, 18, 19]. Liao *et al* [3, 10] have presented the study with the most extended follow-up of 5 years in cT4b OSCC.

### Risk of bias analysis

**Figure 4.**
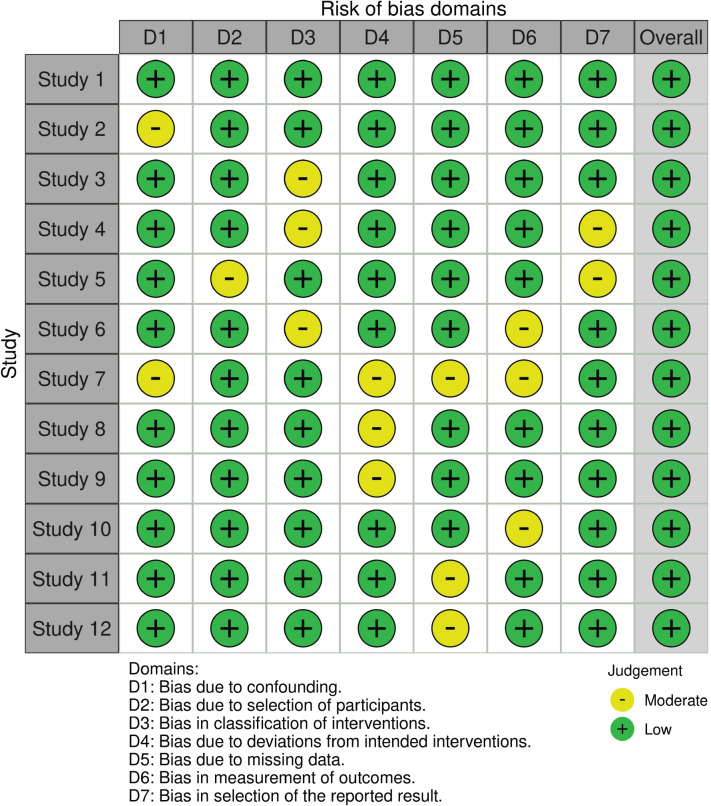
Risk of bias traffic plot.

**Figure 5.**
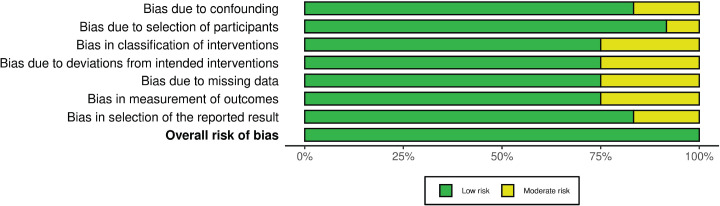
Risk of bias summary plot.

ROBINS-I V2 tool [21] was used to evaluate the risk of bias in included studies in relation to the studied result (i.e., the reported incidence of pathologically N0 lymph nodes). The risk of bias in the traffic plot and summary plot indicates a moderate to low risk of bias, considering the research question regarding the incidence of pathologically negative lymph nodes (Figures 4 and 5).

### Sensitivity analysis

A sensitivity analysis was conducted to investigate the factors contributing to the significant heterogeneity identified in the proportional meta-analysis. Such an analysis was performed by segregating the included studies based on neoadjuvant chemotherapy (NACT) + surgery and Primary surgery Cohorts. The segregation again revealed significant heterogeneity, indicating heterogeneous data within the cohort.

A meta-analysis was conducted on studies that reported compartment resection as the standard operating procedure, resulting in an *I*^2^ value greater than 90%.

The authors of this review identified that non-uniformity of data reporting, inclusion of different subsites, inclusion of both supra- and infra-notch disease and NACT are factors that contribute to high heterogeneity.

## Discussion

Clinico-radiologic cT4b OSCC has been considered an advanced stage of disease with Low overall DFS. There is a current debate regarding the Triage of patients with cT4b OSCC for curative intent surgery.

To address the critical question, a proportional meta-analysis attempted to evaluate the most significant prognostic determinants in these patients, viz, positive neck nodes. The proportional meta-analysis revealed that 40.58% patients with cT4b disease have pathologically negative lymph nodes. This information is critical in light of a recently reported study by Liao *et al* [3], who reported lymph node metastatic positive probability as a significant prognostic determinant. Zanoni *et al* [22] reported pathologically positive nodal status as the most powerful and consistent predicter of survival in patients with OSCC.

A proportional meta-analysis of survival outcomes could not be conducted due to heterogeneous data. However, survival data, as reported in supra-notch T4b disease, are shown in Table 2. It has already been established in literature that infra-notch cT4b disease has a comparative survival outcome to cT4a disease [23].

3-dimensional clearance of disease is the primary requirement of any surgical resection; a single surgical technique has not been standardised for all patients. However, compartment resection has attempted to standardise the surgical technique and extent of excision. It has become a tool for comparing treatment results among various surgical units, with reported improvements in marginal clearance [19]. The treatment benefit achieved in this cohort of patients will define and determine the future resectability of cT4b disease as classified using the TNM AJCC 9th edition.

### Selection criteria for curative intent surgery

The key factors guiding surgeons in choosing curative intent surgery are listed separately in Table 4, which are widely based on the anatomic extent of the disease. The earliest paper by Liao *et al* [10] recommends against surgical resection of supra-notch disease, and Rai *et al* [17] suggest compartmental surgical resection even in cases of supra-notch disease without bone involvement or lymphatic metastasis. However, any supra-notch disease with bone involvement, positive neck nodes, pterygomaxillary fissure involvement or disease above the lateral pterygoid muscle is a critical factor for NACT to evaluate disease biology and for post-NACT reassessment of surgical resectability.

Currently, many authors have classified the disease based on its anatomical extent (Table 5), describing either anterior or posterior disease [10], upper disease and lower disease [17], while most commonly mentioned supra and infra-notch disease [10], which has been correlated with tumour biology and surgical resectability. Kumar *et al* [24] described the T4b disease in terms of the number of muscles involved.

The evidence in favour or against the resectability can only be decided by the survival benefits associated with curative intent surgery. The survival outcomes have been described in Table 3. The heterogeneous nature of data reporting has prevented us from doing proportional meta-analysis of survival benefits.

Such heterogeneity suggests a need to reclassify the disease currently designated as cT4b. The technical challenges have been overcome by utilising compartment resection, which has enabled the achievement of safe margins. However, the term ‘technical challenge’ is not the best description of the non-resectability of this disease, as it has been described to have an aggressive biology. Hence, the survival benefits of aggressive surgical resection of such tumours, when technically possible, need to be highlighted.

Our systematic review highlighted that 40.58% of patients with cT4b disease have pN0 disease. 7 out of 11 included studies would not opt for upfront surgery in a cT4b supra-notch disease. The prognosis is worse when the bone and pterygomaxillary fissure are involved. MRI examination is the most suitable radiographic assessment for surgical planning, as well as for evaluating tumour response to NACT. However, the heterogeneity of imaging modalities used in the included studies also highlights the need for standardisation on imaging protocols.

The clinical and radiographic examinations can only infer tumour biology. The anatomical spread of the disease in T4b OSCC not only suggests a challenging resection but may also indicate aggressive tumour biology, as previously noted by Liao *et al* [3, 10]. The application of tumour biology in disease management has led to the introduction of immunotherapy. While this meta-analysis was ongoing, the FDA approved a new standard of care based on the use of Pembrolizumab, which was previously authorised for use in metastatic or recurrent OSCC.

### Neo-adjuvant Pembrolizumab and the new standard of care

Conventional Standard of care in cT4b oral malignancy is NACT-Sx /Primary surgery-CTRT, which has failed to improve outcomes in the last 2 decades (chances of cure under 50%). Recent primary results from the phase 3 KEYNOTE-689 trial [25]. A study that evaluated a pembrolizumab-based regimen versus adjuvant radiotherapy with or without cisplatin in 714 resectable locally advanced lesions suggested an improved event-free survival with pembrolizumab therapy in PD-L1 positive disease (27% improvement in EFS). Hence, the FDA has recently approved pembrolizumab therapy for locally advanced OSCC.

A recently reported study from Tata Memorial Hospital, Mumbai, by Peelay *et al* [26]. examined the incidence of PD-L1 expression in oral malignancies within the Indian population. The study reports that 90% of patients have a PD-L1 expression (Tumour Proportion Score) of more than 1%, which is significant, as noted in KEYNOTE-689 [25]. Phase 3 trial and recent FDA approval: patients with PD-L1 expression of more than 1% (combined positive score) are selected for both neo-adjuvant and adjuvant therapy with pembrolizumab. This indicates that the majority of oral cancer patients from the Indian population could potentially benefit from this new standard of care.

Challenges persist regarding the affordability of Pembrolizumab therapy in financially constrained populations of developing countries; therefore, the current standard of care remains relevant until immunotherapy becomes affordable or accessible in public health systems worldwide.

## Conclusion

The systematic review identified significant heterogeneity, making a meta-analysis of all tumour-related prognostic markers unfeasible. However, analysis of data on the proportion of patients with N0 lymph nodes reveals that 40% of patients with cT4b disease may be suitable candidates for curative intent surgery if technically resectable. There is a consensus that patients with supra-notch cT4b disease generally have a poor prognosis. Involvement of pterygoid plates and lymph node metastasis are indicators of poor prognosis. The role of newly approved neoadjuvant immunotherapy in improving survival outcomes warrants further study in ongoing and future trials.

## Conflicts of interest

The authors declare that they have no conflicts of interest.

## Funding

The authors declare that no funding was received for the work and publication of this meta-analysis.

## Consent to participate

Not applicable.

## Consent for publication

No person’s data used. It is a systematic review based on available literature.

## Ethical approval

Not applicable.

## Author contributions

Author SP contributed to the conceptualisation of the research and literature search. SP and SoP were involved in data synthesis. Statistical analysis was performed by MP, and manuscript proofreading was done by SR.

## Availability of data and materials

No such data has been shared with any platform.

## Code availability

No software, AI or custom code was used for the preparation of this manuscript.

## Clinical trial registration

Not applicable.

## Figures and Tables

**Table 1.** Search strategy for database search.

Database	Search strategy	No. of results
Ovid discovery	(locally advanced oral cancer) AND t4b AND (oral cancer)	486
Pubmed	((t4b) AND (oral cancer)) AND (locally advanced)	18
Clinical key	t4b oral cancer	100
Pubmed	(compartment resection) AND (oral cancer)	38
Cochrane	t4b oral cancer	37
Embase	(‘mouth cancer’/exp OR ‘mouth cancer’) AND compartment AND resection	22
Embase	‘locally advanced head and neck SCC/exp	18
Scopus	(ALL (locally AND advanced AND head AND neck AND cancer ) AND TITLE-ABS-KEY ( **tab** ) AND ALL (compartment AND resection))	21

Population: Patients with SCC of the oral cavity with cT4b (very advanced oral cancer with involvement of muscles of mastication)?

Intervention: Curative intent surgery (wide local excision/ITF Compartment resection).

Control: Not applicable

**Table 2.** Study characteristics.

	Year and author	Study type	Journal	Age range	T4b	N0 Nodes	Adjuvant CT/RT	NACT	Skin involvement	Clear margins	Reconstruction	Post-op trismus	Local recurrence	DFS	OS
1.	Mohiyuddin *et al*(2019) [20]	R	EAOL	50–60 years	52	0	CT-36 CT &RT- 16	20 (Partial response- 13)	36	31	PMMC-42 ALT-4 FF-6	9/21	14	31 (2 years)	60% (2years)
2.	Pillai *et al* (2019) [18]	P	OO	25–86 years	181	37	CT-66 CT &RT-91	-	35	173	-	-	-	110 (2 years)	58% (2years)
3.	Rai *et al* (2023) [17]	R	IJOMS	21–70 years	162	83	CT&RT-110	70	41	94	-	-	65	56 (5 years)	41.4% (5years)
4.	Gangopadhyay *et al* (2021) [13]	R	L	21–75	302	57	RT- 111	56	13	84	-	-	66	56 (3 years)	46.1% (5 years)
5.	Liao *et al* (2007) [10]	R	OO		45	(pN0-1) - 29	RT/CT&RT-29	-	3 (Infra-notch)	35	-	-	19	Supra-notch- 14.3 (5 years) Infranotch- 64.7 (5 years)	Supra-notch- 14.3 (5 years) Infranotch- 55.3 (5 years)
6	Baddour *et al* (2021) [19]	R	Laryng	36–91 years	25	8	RT-2 CT&RT-16	-	-	1	-	-	9	13 (2 years)	44% (2 years)
7	Trivedi *et al* (2015) [14]	P	IJC	16–18 years	45	28	RT- 41 CT&RT- 8	-	30	42	-	-	7	38 (21 months median)	88.8% (21 months median)
8	Horowitz *et al* (2022) [12]	R	Head and neck	60.3 ± 12.1	132	42	RT-99/RT&CT-22	NA	NA	44	NA	NA	NA	24 months Supranotch – 54.1% Infra-notch – 57.1	NA
9	Brindha *et al* (2022) [11]	R	IJO&HN	60–70 years	20	17	CT &RT- 20	-	-	12	-	-	6	12 (30 months mean)	-
10	Mair *et al* (2018) [15]	P	OO	26–73 years	75	32	NA	-	NA	66	-	-	22	29 (22 months mean)	50.6% (22 months mean)
11	Patel *et al* (2021) [16]	R	HN	24–90		42	RT-99 CT-22	-	-	44	-	-	-	NA	NA

R- Retrospective study, P- Prospective study, CT- Chemotherapy, RT- Radiotherapy, NACT- Neoadjuvant Chemotherapy, PMMC- Pectoralis Major Myocutaneous Flap, ALT- Anterolateral thigh Flap, FF- Forehead Flap, Eur Arch Otorhinolaryngol - European archives of Oto-Rhino-laryngology, OO.- Oral Oncology, IJOMS- International Journal of Oral and Maxillofacial Surgery, Laryng-Laryngoscope, IJC-Indian Journal of Cancer, IJO&HN- Indian Journal of Otolaryngology and Head and Neck Surgery, CO- Clinical Otolaryngology, HN- Head and Neck ( Journal of the sciences and specialities of head and neck)

**Table 3.** Survival outcomes in patients with supra-notch cT4b OSCC.

Author	Supra notch	DFS	OS
Gangopadhyay *et al* [13]	34	39.8% (3 years)	41.2% (3 years)
Pillai *et al* [18]	51	51% (2 years)	40.2 months (mean)
Liao *et al* [10]	7	14.3% (5years)	14.3% (5 years)
Baddour *et al* [19]	6	42% (3 years)	50% (24 months)
Rai *et al* [17]	46	34.8% (5-year)	26.1% (5-year)

**Table 4.** Indications for upfront curative intent surgery.

Authors	T4b resectability
Mohiyuddin *et al* [20]	Compartment resection of ITF - tumours inferior to the lateral pterygoid and involving the anterior half of the masticatory compartment (loco-regional control of 60%)
Pillai *et al* [18]	A curative intent surgical resection is offered to all patients with T4b disease except those with skull base involvement, internal carotid involvement, and patients with poor performance status.
Rai *et al* [17]	Upfront surgery: The **Upper disease** tumours without bone involvement and nodal metastasis have better prognosis. NACT - supra-notch tumours involving MS <1 cm from the skull base. -Skin involvement above the zygomatic arch, -large nodes fixed to vital structures, - mandibular canal involvement with extensive perineural spread not extending beyond the skull base foramina.
Gangopadhyay *et al* [13]	Poor prognosis: Involvement of bone, nodes associated with limited survival outcomes and therefore extensive surgery should be avoided. Upfront surgery: If R0 resection is possible
Liao *et al* [10]	Supra-notch T4b tumours are not unresectable; the tumour excision should target infra-notch tumours.
Baddour *et al* [19]	Unresectable T4b(excluded) - Pterygoid plate involvement, skull base involvement and internal carotid artery encasement.
Trivedi *et al* [14]	Unresectable T4b- involvement of internal or common carotid artery, pre‑vertebral fascia and tumours with intracranial extension. involvement of the pterygomaxillary fissure – Poor prognosis
Kumar *et al* [24]	T4b OSCC with three or fewer masticatory structures are resectable.
Brindha *et al* [11]	Treated supra-notch T4b with compartment resection Disease extending above the lateral pterygoid - Inoperable
Mair *et al* [15]	Upfront surgery: For Infra-notch tumours. NACT and reassessment: for supra-notch tumours. Unresectable: supra-notch tumours with carotid artery encasement and skull base involvement, palliative intent treatment should be considered.
Horowitz *et al* [12]	Unresectable - Skull base involvement, ICA encasement. In Supra-notch disease, worse survival outcomes.

**Table 5.** Various classification systems for cT4b disease.

Supra-notch /Infra notch disease.		
Supra-notch disease disease extending above the sigmoid notch of the mandible. Tumours involving the lateral pterygoid muscle and/or temporalis muscle and/or extending into the retroantral fat space in the ITF and/or pterygoid plates/hamulus.	Infra-notch disease disease extending below the sigmoid notch of the mandible. Tumours involving the medial pterygoid muscle and/or masseter muscle and/or the vertical ramus of the mandible.	
		
**Upper disease/Lower disease.**		
Upper disease - defined as a disease epicentre located above an imaginary plane passing through the inferior border of the retromolar trigone; subsites including the retromolar trigone, buccal mucosa, upper gingivobuccal sulcus, upper alveolus, and hard palate were defined as upper disease.	Lower disease - defined as a disease epicentre located below the inferior border of the retromolar trigone; subsites including the lower gingivobuccal sulcus, tongue, floor of the mouth, and lower alveolus were defined as lower disease.	
		
**ITF involvement [14].**		
Category I: Low ITF – involvement of medial pterygoid and masseter muscle	Category II: Intermediate ITF – involvement of both pterygoid muscles, temporalis muscle, and pterygoid plates	Category III: High ITF – (A) involvement of pterygomaxillary fissure and (B) involvement of intracranial compartment
		
**Anterior space/ posterior space (divided by a vertical plane crossing mid-portion of the ramus).**		
Anterior space tumors- involving structures only anterior to the vertical plane such as anterior half of the masseter/ or pterygoid muscles	Posterior space tumors- involves tumors that cross the vertical place posteriorly to involve structures such as posterior half of the masseter /pterygoid muscles	
